# Expression of the HPV16E7 Oncoprotein by Thymic Epithelium is Accompanied by Disrupted T Cell Maturation and a Failure of the Thymus to Involute with Age

**DOI:** 10.1080/10446670310001626562

**Published:** 2003

**Authors:** K. M. Malcolm, J. Gill, G. R. Leggatt, R. Boyd, P. Lambert, I. H. Frazer

**Affiliations:** 1 Centre for Immunology and Cancer Research University of QLD Princess Alexandra Hospital Ipswich, Road Buranda Qld. 4170 Australia; 2 Department of Pathology and Immunology Monash University Medical School Commercial Road Praharan VIC. 3181 Australia; 3 McArdle Laboratory for Cancer Research University of Wisconsin medical School Madison WI 53706 USA

## Abstract

Transgenic mice expressing the E7 protein of HPV16 from the keratin 14 promoter demonstrate increasing thymic hypertrophy with age. This hypertrophy is associated with increased absolute numbers of all thymocyte types, and with increased cortical and medullary cellularity. In the thymic medulla, increased compartmentalization of the major thymic stromal cell types and expansion of thymic epithelial cell population is observed. Neither an increased rate of immature thymocyte division nor a decreased rate of immature thymocyte death was able to account for the observed hypertrophy.

Thymocytes with reduced levels of expression of CD4 and/or CD8 were more abundant in transgenic (tg) mice and became increasingly more so with age. These thymic SP and DP populations with reduced levels of CD4 and/or CD8 markers had a lower rate of apoptosis in the tg than in the non-tg mice. The rate of export of mature thymocytes to peripheral lymphoid organs was less in tg animals relative to the pool of available mature cells, particularly for the increasingly abundant CD4^lo^ population. We therefore suggest that mature thymocytes that would normally die in the thymus gradually accumulated in E7 transgenic animals, perhaps as a consequence of exposure to a hypertrophied E7-expressing thymic epithelium or to factors secreted by this expanded thymic stromal cell population. The K14E7 transgenic mouse thus provides a unique model to study effects of the thymic epithelial cell compartment on thymus development and involution.


*Clinical & Developmental Immunology*, June–December 2003 Vol. 10 (2–4), pp. 91–103

Taylor & Francis healthsciences

### Expression of the HPV16E7 Oncoprotein by Thymic Epithelium is Accompanied by Disrupted T Cell Maturation and a Failure of the Thymus to Involute with Age

K.M. MALCOLM[a], J. GILL[b], G.R. LEGGATT[a], R. BOYD[b], P. LAMBERT[c] and I.H. FRAZER[a,*]

[a]*Centre for Immunology and Cancer Research, University of QLD, Princess Alexandra Hospital, Ipswich, Road, Buranda, Qld., 4170 Australia;* [b]*Department of Pathology and Immunology, Monash University Medical School, Commercial Road, Praharan, VIC., 3181 Australia;* [c]*McArdle Laboratory for Cancer Research, University of Wisconsin medical School, Madison, WI 53706, USA*

Transgenic mice expressing the E7 protein of HPV16 from the keratin 14 promoter demonstrate increasing thymic hypertrophy with age. This hypertrophy is associated with increased absolute numbers of all thymocyte types, and with increased cortical and medullary cellularity. In the thymic medulla, increased compartmentalization of the major thymic stromal cell types and expansion of thymic epithelial cell population is observed. Neither an increased rate of immature thymocyte division nor a decreased rate of immature thymocyte death was able to account for the observed hypertrophy.

Thymocytes with reduced levels of expression of CD4 and/or CD8 were more abundant in transgenic (tg) mice and became increasingly more so with age. These thymic SP and DP populations with reduced levels of CD4 and/or CD8 markers had a lower rate of apoptosis in the tg than in the non-tg mice. The rate of export of mature thymocytes to peripheral lymphoid organs was less in tg animals relative to the pool of available mature cells, particularly for the increasingly abundant $CD4^{lo}$ population. We therefore suggest that mature thymocytes that would normally die in the thymus gradually accumulated in E7 transgenic animals, perhaps as a consequence of exposure to a hypertrophied E7-expressing thymic epithelium or to factors secreted by this expanded thymic stromal cell population. The K14E7 transgenic mouse thus provides a unique model to study effects of the thymic epithelial cell compartment on thymus development and involution.

*Keywords*: Thymus; T lymphocyte; Cellular differentiation; Transgenic

*Abbreviations*: tg, transgenic; TN, triple negative; DP, double positive; SP, single positive; HSA, heat shock antigen; TEC, thymic epithelial cells; K5, keratin 5; K14E7, FVB mice transgenic for the E7 protein of Human Papillo ma virus type 16 driven off the keratin promoter; BrdU, 5-bromo-2′-deoxyuridine; PI, propidium iodine; LN, lymph node; AAD, amino actinomycin D

### INTRODUCTION

T cell precursors first enter the cortex of the thymus from the bone marrow and subsequently go through four discrete developmental stages within the $CD4^-CD8^-CD3^-$ triple negative (TN) T cell precursor population. The most immature TN thymocytes (TN1), defined by their $CD44^+CD25^-$ staining pattern, give rise to the TN2 $CD44^+CD25^+$ subset. TCR rearrangements begin at this stage and once the cells pass to the TN3 ($CD44^-CD25^+$) stage they are committed to the T cell lineage. Following downregulation of CD25 in the final TN4 stage, they develop into the predominant thymic population which expresses neither CD25 nor CD44 and is double positive (DP) for the expression of CD4 and CD8. Further rearrangement occurs and the DP cells move into the medullary area as either $CD4^+$ single positive (SP) or $CD8^+$ SP. During their time in the medulla there is modification of additional markers such as CD69, heat shock antigen (HSA), L-selectin and CD45RB, as they await export to the periphery (Gabor *et al.*, 1997b).

Interplay between thymocytes, thymic epithelium and other cells of the thymic stroma regulate both thymic development and T cell maturation. Signals provided by the thymic epithelium regulate the passage of thymocytes through developmental checkpoints. Thymic involution occurs in the maturing animal, and is associated with a progressive loss of thymus cortex and normal thymic

*Corresponding author. Address: Centre of Immunology and Cancer Research, Level 4, R Wing, Princess Alexandra Hospital, Ipswich Road, Buranda, QLD, 4102, Australia. Tel.: +61-7-3240-5315. Fax: +61-7-3240-5946. E-mail: ifrazer@cicr.uq.edu.au

ISSN 1740-2552 print/ISSN 1740-2530 online © 2003 Taylor & Francis Ltd
DOI: 10.1080/10446670310001626562

architecture, with a decline in the output of mature T cells. With increasing age, there is also a decline in the capacity of the thymus to support T cell differentiation, the composition of the peripheral T cell compartment changes, and there is a loss of immune function (Utsuyama *et al.*, 1991; Thoman, 1995).

Disturbance of the stromal or T cell components of the thymus by targeted transgene expression has been used previously to analyze the factors controlling thymus and thymocyte development. In the model presented here, targeted expression of the human papillomavirus 16 oncogenic E7 protein to basal epidermal keratinocytes has produced an animal in which the thymus fails to involute and becomes progressively hypertrophic with age. These mice have relatively normal antibody and T helper cell responses to E7 and other antigens and have no evidence of autoimmunity to the E7 expressed in their thymus or skin (Frazer *et al.*, 1998). A reduced $CD8^+$ E7-specific and non-E7 specific $CD8^+$ CTL response is the only immunological consequence of expression of their transgene reported to this point (Tindle *et al.*, 2001).

Other tg mice exhibiting thymic hypertrophy include the K5-cyclin D1 and the K5 HPV16E6/E7 tg mice. An expanded cortical thymic epithelial cell (TEC) precursor compartment in the cyclin D1 mice provides an increased availability of developmental niches for maturing pre-T cells (Klug *et al.*, 2000). An increased rate of early thymocyte cycling leads to an expanded thymus containing a normal thymocyte subset distribution. Similarly, the deregulation of epithelial cell proliferation through the expression of HPV16 E6/E7 in the keratin 5 (K5) precursor epithelial compartment of the recently described K5 HPV16E6/E7 tg mice did not alter the outcome of T cell differentiation (Carraresi *et al.*, 2001). The observed normal T cell development in both strains of tg mice was attributed to the continuing expansion of a thymic niche supportive of early stage T cell development. In contrast, the Human CD3e transgenic mice, in which the minor cortical subset $K8^+K18^+K5^+K14^-$ is the predominant epithelial subset, develop a poorly organized, primitive thymus in which thymocyte development is inhibited at the $CD44^+CD25^-$ pro T cell stage (Hollander *et al.*, 1995). The lack of developmental niches for the later stages of T cell maturation resulting in a very small thymus lacking $CD4^+$ and $CD8^+$ SP thymocytes. K14 is a keratin isoform selectively expressed in mature thymic epithelium. By analogy with the effects of E7 as a transgene in other cell types, expression of the oncogenic E7 protein in the thymic epithelium of the K14E7 mice would likely induce a selective expansion of this mature $K14^+$ TEC compartment. Such an expansion may produce a thymus more able to support late stage T cell development. A study of the T cell developmental in a mouse in which the thymic microenvironment is altered in this way may thus shed further light on the effect of stromal cell composition upon T cell maturation and thymic function.

### MATERIALS AND METHODS

### Mice

FVB and FVB mice transgenic for the E7 protein of Human Papilloma virus type 16 driven off the keratin promoter (K14E7) (Herber *et al.*, 1996) were obtained from the Animal Research Centre, Perth, and were housed under specific pathogen free conditions.

### Measurement of E7 mRNA

HPV16 E7 mRNA was measured by real time polymerase chain reaction of reverse transcriptase generated cDNA using the Perkin Elmer Taqman system as described elsewhere (Frazer *et al.*, 2001). Briefly, PCR of 150 ng of sample mRNA was performed using E7 specific primers and E7 mRNA estimations based on a standard curve derived from serial dilutions of plasmid pHPV16.

### Histology

Gross thymic architecture was assessed by staining slides made from paraffin-embedded thymii of 6 month old FVB and K14E7 mice with hematoxylin and eosin. Thymii from mice of this age were also snap frozen in O.C.T. Compound (Tissue-Tek, Sakura, Tokyo) for later cryostat processing. These mice had been injected intraperitoneally with 100 mg/kg body weight 5-bromo-2′-deoxyuridine (BrdU) (Sigma) in PBS at 0 and 4 h and sacrificed at 5 h. Five micrometer sections were cut and stained with FITC labeled anti -BrdU (Pharmingen) in addition to anti-K14, -K8, -CD3,-MTS 7 and MTS 15 (as described below). Sections were stained as a two or three step process. Anti-keratins were biotinylated and therefore required streptavidin conjugated-alexa-488 (green), -cy-5 (blue) or -alexa 568 (red) as tertiary antibodies. CD3 and antigens of the MTS series were detected in a two step process using rat primary antibodies and anti rat-alexa-488, -alexa 568 and -cy-5 as above.

### Cell Suspensions

Single cell thymic preparations were made by following a 30 min digestion in a 1 mg/ml solution of Collagenase A (Boehringer Mannheim) in PBS at 37°C. Red blood cells were remove d from both thymic and lymph node (LN) preparations following incubation in 1 ml ACK lysis buffer (0.15 M $NH_4Cl$, 1 M $KHCO_3$, 0.1 mM EDTA) for 2 min at room temperature, followed by a wash in ice cold PBS-FCS 1%.

### Flow Cytometry

Single cell suspensions of thymic and LN cells were stained with mAbs as described in Rodewald *et al.* (1995). Data on $10^5$ cells per test were collected using a Fascalibur (Becton Dickinson, Mountain View, CA) and analyzed using CellQuest (Becton Dickinson) software. In situations were the number of cells in a particular gate were very small, the total number of cells collected per test was increased in order to give an accurate measurement. Analyzed cells were selectively gated on forward light scatter versus side light scatter to eliminate dead cells and debris from analysis. An EPICS Elite ESP flow cytometer (Coulter, Hiateah, FL, USA) was used to separate individual cell populations based upon their CD25/CD44 or CD4/CD8 staining patterns.

### Antibodies

Unless otherwise noted, all antibodies were purchased from PharMingen (San Diego, CA). For flow cytometric analysis the following antibodies were used: FITC-labeled CD25, biotinylated CD44/ PerCP labeled Streptavidin, CD3 APC and CD11b APC, PE labeled CD4, biotinylated CD8/ APC labeled Streptavidin, PerCP labeled CD3, anti-BrdU-FITC, biotinylated CD3/ PerCP labeled streptavidin, APC labeled CD8, PE labeled CDllb, and APC labeled CD25. Thymic emigration rate experiments used FITC isomer 1 (for intrathymic injection, purchased from Becton Dickinson). Rabbit antisera specific for mouse K14 (Roop *et al.*, 1984) was obtained from Covance Research (Richmond, CA). Thymic stromal antibodies of the MTS series, prepared as rabbit polyclonal serum, have been described elsewhere (Godfrey *et al.*, 1990; Gray *et al.*, 2002). TN thymocytes were selectively analyzed by excluding cells which stained positive for a cocktail of FITC- labeled antibodies which included anti-Gr1, -Mac-1, -CD3, -CD4, -CD8 and -B220.

### Intrathymic Labeling

The technique us ed to FITC label thymocytes has been described elsewhere (Berzins *et al.*, 1999). Each lobe was injected with approximately 10 μl of 350 μg/ml FITC. Mice were killed by $CO_2$ asphyxiation 24 h postinjection and thymii, LN and spleen taken for analysis. The migration of thymocytes to the periphery was determined by calculating the number of FITC positive cells in the peripheral lymphoid organs 24 h following intrathymic labeling as a fraction of the total number of thymocytes labeled (Scollay *et al.*, 1980).

### BrdU Measurement of Proliferation

Mice were injected intraperitoneally with BrdU as above. Thymus, spleen and peripheral LN were then removed into PBS-FCS 1%. Cell suspensions of these organs were counted, washed and stained for FACS for differential analysis of the proliferation of individual subpopulations. Following staining for cell surface markers, each test was resuspended in 100 μl PBS-FCS 1%. One milliliter of 1% paraformaldehyde was added dropwise to this suspension and the cells were then incubated overnight at 4°C. The following day the cells were washed twice in PBS-FCS 2% and permeabilized using 50 ml of a 0.0001% DNAase in digestion buffer (2 M NaCl, 4.3 mM $MgCl_2$). Cells were incubated in a 37°C water bath for 30 min and subsequently washed three times in PBS-FCS 2%. 40 μl of anti-BrdU-FITC (at 1:2 in PBS-FCS 5–0.5% Tween-20) was added to the washed cell pellet and incubated for 30 min at RT. Following two washes in PBS-FCS 2% the cells were resuspended in 200 μl wash buffer and analyzed by FACS.

TUNEL analysis of apoptosis in whole tissue thymic preparations: an *in situ* cell death detection kit (TUNEL) (Boehringer Mannheim) was used according to the manufacturer's instructions.

### TEC Enrichment

Thymic cell preparations were enriched for epithelium by injecting the mice intraperitoneally 48 h prior to thymus harvest with 0.5 mg cortisone per 20 g mouse (Dexamethasone sodium phosphate, David Bull Laboratories). The thymic cell preparations were prepared as described previously (Schreiber *et al.*, 1991) and then cultured overnight in 1.35 M deoxyguanosine to further deplete the preparations of T cells.

### Apoptosis within Cell Subpopulations

Cells which had been stained with the anti- CD25, -CD44, -CD4 and -CD8 Mabs as described above were subsequently stained with a 7-amino-actinomycin D (7-AAD) solution (Viaprobe, BD PharMingen), used according to the manufacturers instructions. This allowed a determination of the dead and dying cells within the subpopulations.

### Cell Cycle Analysis

Fresh thymocytes were stained for surface marker expression in the usual way and then stained with propidium iodide (PI) according to the method outlined in Current Protocols in Immunology (1999). Data was collected on the FACScalibur in linear mode for cell cycle analysis.

### RESULTS

### The K14E7 Mouse Thymus Fails to Involute Post-puberty

The Human Papillomavirus type 16 E7 gene is driven off a keratin 14 promoter in a line of K14E7 transgenic FVB mice (Herber *et al.*, 1996). Using Real-Time PCR, E7 mRNA could be detected in these animals in skin, thymus, upper digestive tract and stomach. The skin and thymus had 8.3 ± 0.5 × 10 5 and 7.2 ± 0.5 × 10 5 copies of E7 mRNA per ng total mRNA, respectively.

Compared with non-transgenic FVB littermates, older K14E7 tg mice exhibited thymic hypertrophy. Failure of the K14E7 tg mouse thymus to undergo the normal

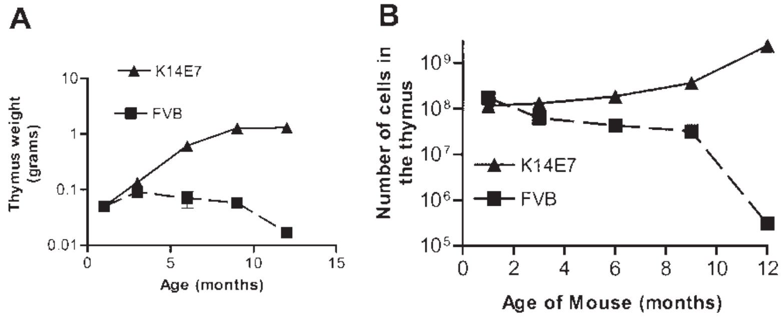

FIGURE 1 Failure of the K14E7 mouse thymus to involute with age. Thymic weight (A) and cellularity (B) are shown for K14E7 tg and non-tg FVB thymus. Weights and cell counts are the means ± SEM for 4–5 mice at each age.

involution which accompanies ageing was followed by increased thymic weight and cellularity (Fig. 1A,B). Thymii of more than 1 g and $10^9$ cells were observed in some 12 month old animals. The absolute number of cells per spleen or LN was approximately 1.5 times greater in the adult K14E7 mice than in non-transgenic FVB mice (from 3 months of age).

Thymic hypertrophy similar to that observed in K14E7tg FVB mice was observed in C57Bl/6 mice expressing the same transgene construct. The effects of E7 on cell growth and differentiation are abolished by mutations at the CKII site (Davies *et al.*, 1993; Demers *et al.*, 1996) and mice transgenic for a mutant E7 lacking a functional CKII site, driven from the K14 promoter did not develop enlarged thymii. Failure of involution, and subsequent hypertrophy of the thymus of the K14E7 tg mouse, is thus likely to be a consequence of the production of biologically active E7 in the thymus.

### Expression of the E7 Transgene Alters Thymic Architecture

Expression of a K14-controlled transgene would be expected to be most evident in the thymic medulla area where K14+ epithelial cells are found (Klug *et al.*, 1998; Burns *et al.*, 1999), though it is noted that transgenes driven from the K14 promoter are sometimes expressed ectopically in the cortical epithelium (McGargill *et al.*, 2000). Gross architectural changes in the K14E7 tg mouse thymus were, evident in both cortical and medullary areas (Fig. 2A,B). K14E7 tg lobes were more cellular than were non-tg lobes, with the cortical regions significantly expanded. Medullary thymocytes especially were much more densely arranged (Fig. 2A,B, inset).

### Reduced Levels of Expression of CD4 and CD8 on K14E7 tg Thymocytes

The $CD4^+CD8^+$ (DP) and $CD4^+CD8^-$ or $CD4^-CD8^+$ (SP) populations are represented in relatively normal proportions in K14E7 tg mice (Fig. 3). Further, the ratio of mature SP CD4 to SP CD8 T cells was similar for FVB and K14E7 mice, in both the thymus and the LN (data not shown). Reduced levels of CD4 and CD8 expression per cell was observed within the SP and DP populations in the K14E7 tg thymus from 6 weeks of age, becoming more apparent in older mice (Fig. 3), particularly in the $CD3^{hi}$ population. Reduced CD4 and CD8 expression levels were

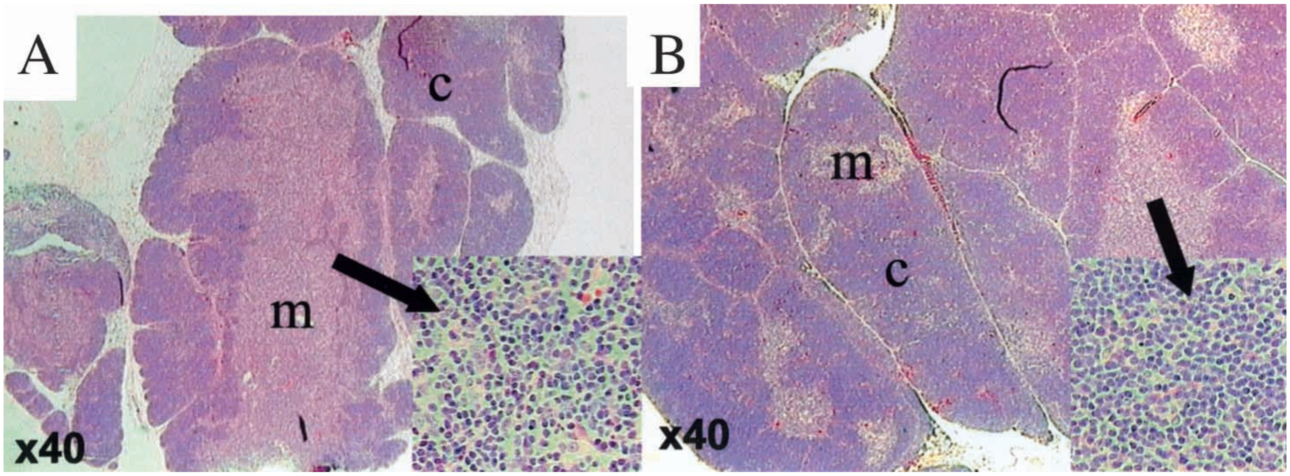

FIGURE 2 Expression of the HPV16E7 transgene in the K14E7 thymus causes gross architectural changes. Hematoxylin and eosin stain of formaldehyde-fixed sections from 6 month old FVB (A) and K14E7 (B) thymii. Expanded cortical regions (c) in the K14E7 thymus are accompanied by more densely packed medullary regions (m) as shown in expanded magnifications of medullary areas at the arrowheads (Magnification, ×400).

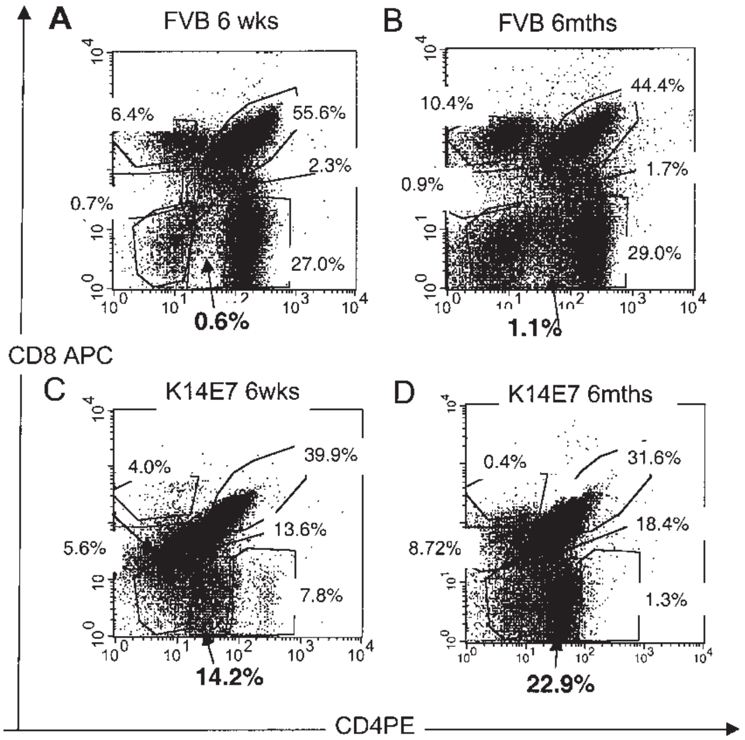

FIGURE 3 CD4 and CD8 expression on E7tg thymic T cells is lower and the number of CD4$^{lo}$ and CD8$^{lo}$ cells increases with age. Thymic cell preparations were from 6 week old (A and C) and 6 month old (B and D) FVB and K14E7 mice were stained with CD4 PE and biotinylated CD8/Streptavidin APC. These figures shown the staining for four individual mice and are representative of four repeats of this experiment.

also observed in peripheral lymphoid organs in older mice (Table I).

### The Cells with Lower Levels of CD4 and CD8 Expression have passed through Selection

CD69 surface expression was more frequent on DP thymocytes in K14E7 tg (7.5 ± 1.4%) than in FVB (4.18 ± 0.4%) animals. Also, more DP cells expressed high levels of CD5 in tg mice (91.0 ± 6.3%, mean level of expression 221.1) than did non-tg animals (61.3 ± 5.4%, mean level of expression 123.2). As CD69 and subsequently CD5 are upregulated on DP cells following positive selection (Gabor *et al.*, 1997a), the higher level of expression of CD69 and CD5 in the K14E7 tg thymus suggested that a greater proportion of the DP cells in tg than the non-tg thymus have passed through selection. There was no difference in the level of TCR expression shown between FVB and K14E7 thymocytes.

### The K14E7 Mouse Thymus is not Enlarged due to an Increased Percentage of Cells in Cycle

Expression of functional but not mutated E7 was associated with thymic hypertrophy, and E7 expression directly increases the fraction of cells in cycle. Although the K14 promoter is not generally active in lymphocytes, and E7 mRNA was not detectable in BM derived cells in the K14E7 tg mouse, low level ectopic E7 expression in thymocytes might nevertheless expand the thymocyte pool. However, incorporation of BrdU was observed in a greater percentage of FVB than of K14E7 thymocytes, BrdU incorporation was less in the CD4$^{lo}$ population than in the CD4$^{hi}$ population in the K14E7 thymus, and less in K14E7 CD4$^{lo}$ thymocytes than in FVB CD4$^{lo}$ thymocytes (Table II). Thus, the increased cellularity and relative preponderance of CD4$^{lo}$ thymocytes in the K14E7 thymus could not be attributed to increased replication amongst immature thymocytes.

TABLE I Expression of CD4 and CD8 on LN cells expressing high levels of CD3

| Mouse strain and age | CD4$^{hi}$ | CD4$^{lo}$ | CD8$^{hi}$ | CD8$^{lo}$ | DP$^{hi}$ | DP$^{lo}$ |
|---|---|---|---|---|---|---|
| FVB 6 weeks | 75.6 ± 3.0 | 0.5 ± 0.0 | 17.6 ± 2.5 | 0.1 ± 0.0 | 1.9 ± 0.1 | 0.2 ± 0.0 |
| K14E7 6 weeks | 78.7 ± 5.5 | 0.1 ± 0.0 | 15.9 ± 2.4 | 0.0 ± 0.0 | 0.7 ± 0.1 | 0.1 ± 0.0 |
| FVB 6 months | 72.7 ± 4.2 | 4.4 ± 0.0 | 14.6 ± 3.3 | 0.1 ± 0.0 | 0.6 ± 0.0 | 0.3 ± 0.1 |
| K14E7 6 months | 61.9 ± 7.5 | 12.4 ± 0.5 | 8.7 ± 0.7 | 0.9 ± 0.1 | 2.3 ± 0.1 | 0.8 ± 0.1 |

Expressed as % positive cells, values are the mean ± 1 S.D. calculated from three separate experiments.

TABLE II BrdU incorporation rates amongst CD3+ thymocytes

| Cell population | $FVB_{6\ weeks}$ | $FVB_{6\ months}$ | $K14E7_{6\ weeks}$ | $K14E7_{6\ months}$ |
|---|---|---|---|---|
| $CD4^{lo}$ | 11.8 ± 0.0 | 43.6 ± 2.2 | 0.6 ± 0.2 | 0.8 ± 0.1 |
| $CD4^{hi}$ | 1.0 ± 0/1 | 5.2 ± 0.3 | 2.1 ± 0.3 | 2.6 ± 0.0 |
| $DP^{lo}$ | 0.6 ± 0.1 | 13.5 ± 1.2 | 0.1 ± 0.0 | 0.3 ± 0.0 |
| $DP^{hi}$ | 0.7 ± 0.1 | 1.9 ± 0.2 | 0.6 ± 0.0 | 0.9 ± 0.1 |
| Whole thymus | 0.9 ± 0.1 | 0.2 ± 0.2 | 0.1 ± 0.0 | 0.2 ± 0.1 |

Mice were given BrdU through ip injection 1 and 5 h prior to thymus harvest. BrdU incorporation was expressed as % population BrdU FITC +ve, calculated as the mean ± 1 S.D. from three separate mice per age and per strain. Thymocytes were triple stained with CD3, CD4 and CD8 and the only the CD3 positive cells used for analysis. The low representation of the CD8 population within the K14E7 thymus made a comparison of BrdU incorporation rates for this population unreliable, the results have therefore not been included.

### There is a Lower Proportion of Cells Undergoing Apoptosis in K14E7 Tg Thymocytes with Downregulated CD4 or CD8

We used 7AAD dye exclusion to examine the rate of cell death in the K14E7 tg T cell populations. Since there were a larger number of total cells in the K14E7 thymus, it is not surprising that there were a significantly higher number of thymocytes in the K14E7 than in the FVB mice incorporating AAD (Fig. 4A). Thymocytes with lower levels of CD4 and CD8 expression clearly have a higher level of apoptosis in both tg and non-tg animals (Fig. 4A). The percentage of cells incorporating AAD in $CD4^{lo}$ and $CD8^{lo}$ populations was, however, lower in the tg than the non-tg thymus. Further, a greater proportion (1.5 times) of $CD4^{lo}$ than $CD4^{hi}$ cells were in G0/G1 in the K14E7 thymus (Fig. 4D). This was not the case for the FVB mouse where the majority of $CD4^{lo}$ cells were undergoing apoptosis, a lesser proportion of cells being in the resting G0/G1 phase. This is especially significant since the $CD4^{lo}$ population accumulates dramatically in K14E7 mice (Fig. 3). Thus, the accumulation of cells in the K14E7 tg thymus is due, at least in part, to a reduced level of cell death in a resting $CD4^{lo}$ compartment.

The lower levels of incorporation of AAD in the K14E7 subpopulation was not reflected in the level of incorporation in the whole K14E7 thymus, which was higher than that shown by the whole FVB thymus. This is explained by AAD corporation studies on TEC enriched thymic preparations which show that K14E7 TEC populations incorporate AAD at a significantly higher rate than do TEC populations from FVB thymii (Fig. 4C).

### The K14E7 Thymus shows a Reduced Rate of Exit to the Periphery for some Subpopulations of T Cells

Intrathymic labeling of tg and non-tg mice with fluorescein followed by analysis of T cells in peripheral lymphoid organs at 24 h was used to estimate exit rates for thymocytes and selectivity of emigration to the periphery (Scollay *et al.*, 1980; Berzins *et al.*, 1999). Intrathymic labeling resulted in between 25 and 65% FITC positive thymocytes. Accumulation in the LN of recent thymic emigrants of all classes was reduced in the K14E7 tg mouse when compared with non transgenic animals (Table III). Although the $CD4^{lo}$ population was one which became progressively larger in the tg thymus with age, the peripheral accumulation of $CD4^{lo}$ cells from the K14E7 thymus occurred at a lower rate in the observed 24 h period than accumulation of other cell types. We therefore postulate that the accumulation of T cells in the K14E7 tg thymus occurs as a result of enhanced survival of a post selection $CD4^{lo}$ T cell population, together with a block to their exit to the periphery.

In support of this, the rate of T cell emigration in K14E7 tg animals, relative to the size of the thymus decreased with age. While the absolute number of recent thymic emigrants accumulating in the peripheral LN was higher in the older mice, the exit of cells expressing normal levels of CD4 and CD8 was 3 fold higher than for $CD4^{lo}$ and $CD8^{lo}$ cells.

### The Earliest Stage of PreT Cell Development is more Highly Represented in the K14E7 tg Thymus

In the 3 month old transgenic thymus, TN1 cells were relatively overrepresented with a correspondingly reduced late pre T cell ($TN4{-}CD3^-4^-8^-44^-25^-$) compartment. This difference was less obvious in older mice, reducing to approximately 10% more TN1 and 10% fewer TN4 in the K14E7 thymus.

CD44 and CD25 expression was similar on $CD3^-CD4^-CD8^-$ pre T cells in young K14E7 tg and non-tg FVB mice. From 12 months of age, however, CD44 expression levels were lower in the TN1 ($CD3^-4^-8^-44^+25^-$) and TN2 ($CD3^-4^-8^-44^+25^+$) subpopulations of tg mice (Fig. 5B,C) than in non-transgenic animals. C-kit expression was reduced in the TN1 cells of K14E7 tg mice, mean expression for FVB TN1 cells (whether expressing $CD44^{hi}$ or $CD44^{lo}$) was 20.1 ± 0.2%, the expression in K14E7 TN1 varied from 13.8 ± 0.4 % for those $CD44^{hi}$ $CD25^-$ to 7.7 ± 0.2 for $CD44^{lo}$ $CD25^-$ TN1 cells Fig. 6.

### The Overrespresented TN1 Population has a Higher Level of Apoptosis

The transition from TN1 into TN2 cells has been shown previously to be accompanied by an approximately fivefold increase in the percentage of cells in cycle

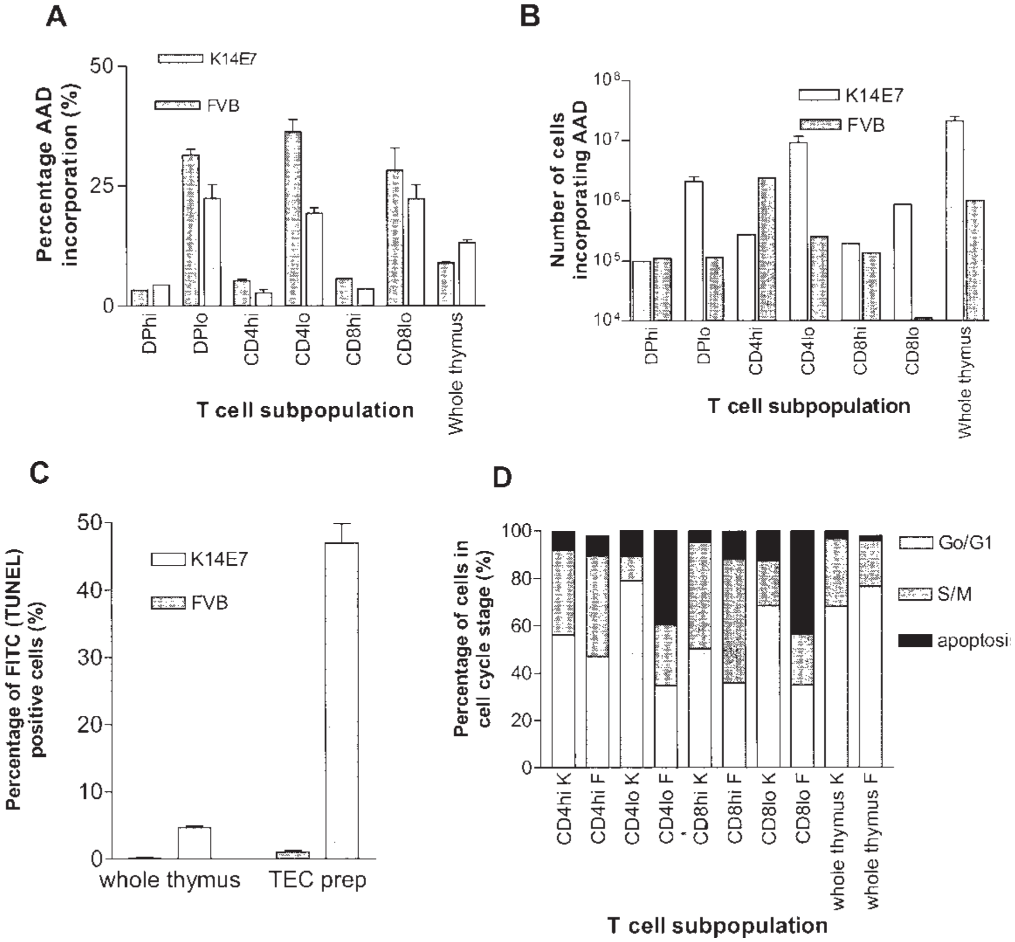

FIGURE 4 Apoptosis is increased in the K14E7 CD4+ and CD8 + thymic populations. (A and B) Thymic cell preparations from FVB and K14E7 mice were stained with biotinylated CD4/Streptavidin APC and CD8 FITC and then incubated in an AAD staining solution. The AAD staining of populations expressing different levels of CD4 and CD8 were compared to determine whether marker downregulation was reflected in a change in the level of apoptosis in the tg mice and is shown as (A) Percentage of AAD incorporation and (B) Absolute numbers of AAD positive cells. Cells were gated on CD4 and $CD8^{lo}$ and $CD8^{hi}$ populations as shown in Fig. 3 to provide estimates of apoptosis in the populations expressing different levels of these surface markers. (C) Single cell suspensions of K14E7 and FVB whole thymii or enriched TEC preparations were stained using a TUNEL assay to examine the contribution made by thymic epithelia to the overall level of apoptosis shown in whole thymus preparations. (D) Cell cycle status of different thymic subpopulations was compared between the K14E7 (K) and non-transgenic FVB (F) mice by staining thymic cell preparations using anti-CD4 and -CD8 surface marker antibodies followed by propridium iodide staining of nuclear DNA. All data shown are the mean results for four mice assayed separately. Three month old mice were used throughout.

TABLE III Migration of FITC-labeled thymocytes to the inguinal LN in 3 month old mice

| | | A | B | C | A × B/C | D | A × B/C/D × 100 |
|---|---|---|---|---|---|---|---|
| Mouse | Cell of interest | % $F^+$ in LN (Mean ± S.E.) | Number of cells of interest per LN ( × $10^5$) (Mean ± S.E.) | Labeling of cell of interest in thymus (%) | Number of $F^+$ cells of interest in LN at 24 h | Number of cells of interest in thymus ( × $10^6$) | Rate of accumulation of (or presence of) $F^+$ migrant population in LN at 24 h relative to potential emigrants population in thymus (%) |
| FVB | $CD4^{lo}$ | 0.51 ± 0.08 | 0.44 ± 0.18 | 33.75 ± 3.10 | 657 | 0.05 ± 0.01 | 1.12 |
| K14E7 | $CD4^{lo}$ | 0.31 ± 0.15 | 1.96 ± 0.31 | 34.83 ± 9.28 | 1740 | 3.77 ± 2.82 | 0.04 |
| FVB | $CD4^{hi}$ | 0.45 ± 0.09 | 9.0 ± 2.60 | 32.21 ± 6.35 | 12 600 | 4.41 ± 2.02 | 0.29 |
| K14E7 | $CD4^{hi}$ | 0.67 ± 0.31 | 11.4 ± 5.70 | 39.71 ± 12.40 | 19 200 | 23.70 ± 12.80 | 0.08 |
| FVB | All T cells | 0.42 ± 0.07 | 20.0 ± 1.00 | 45.15 ± 2.70 | 18 600 | 15.20 ± 8.50 | 0.12 |
| K14E7 | All T cells | 0.41 ± 0.13 | 27.60 ± 1.70 | 45.60 ± 3.38 | 24 800 | 162.0 ± 89.0 | 0.02 |

Calculations are expressed as the Mean ± S.E. for 5–6 mice per strain.

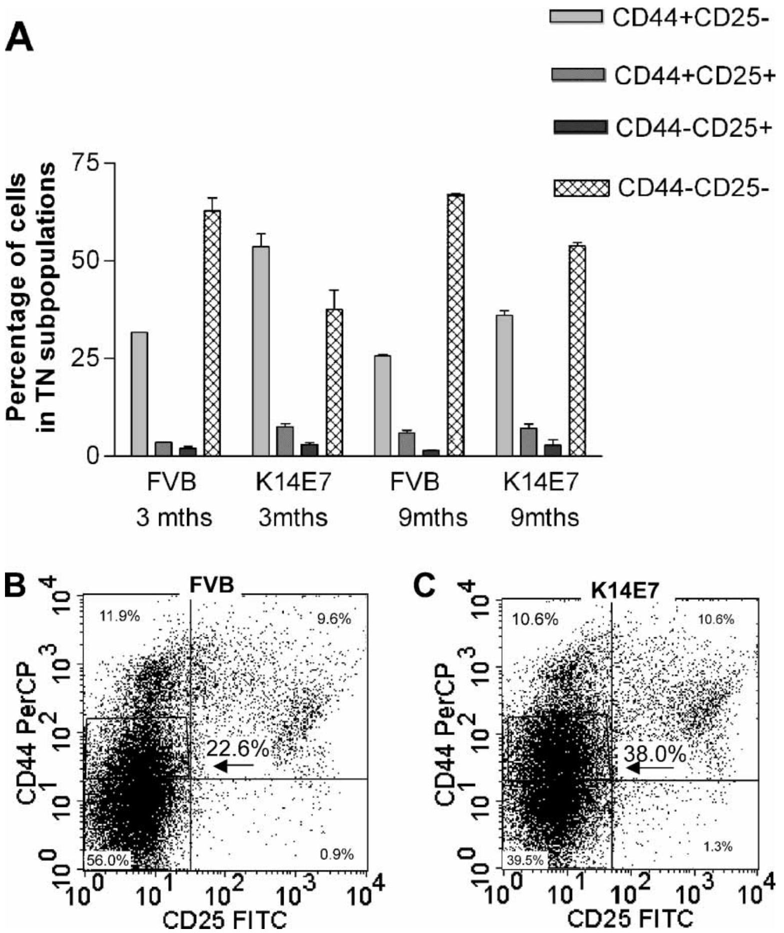

FIGURE 5 Increased CD44 + CD25− (TN1) cells in the K14E7 tg thymus. Collagenase digested thymii were stained with anti-CD44 PerCp, anti-CD25 FITC, anti-CD11b APC and anti-CD3 APC. CD11b and CD3 positive cells were gated out to exclude CD44 + macrophages and CD44+ CD3+ activated T cells. (A) The distribution of TN cells amongst the four subpopulations were compared between young (3 month old) and old (9 month old) non-tg and tg mouse thymii. (B and C) Cell-surface marker expression of CD25 and CD44 within the TN compartment of 9 month old FVB and K14E7 mice. The data shown in these two figures are for a single FVB and a single K14E7 mouse representative of four experiments performed in the same way.

(Penit *et al.*, 1995). When the TN compartment was stained for CD44 and CD25 expression followed by PI incorporation, cell cycle status of the TN stages could be determined. More cells in $G_0/G_1$ were observed amongst the $CD44^{lo}$ TN1 cells in transgenic animals (14.54 ± 1.1%) than non-transgenic animals (3.57 ± 0.3%), with only 1.19 ± 0.12% of K14E7 $CD44^{lo}$ TN1 cells being in the actively dividing $G_2$/M phase compared to 10.19 ± 0.5% of $CD44^{lo}$ TN1 cells in the non-tg mice. A greater percentage of K14E7 TN1 cells were undergoing apoptosis (48.16 ± 4.7% in the K14E7 tg TN1 as compared to 27.11 ± 2.5% in the non-tg TN

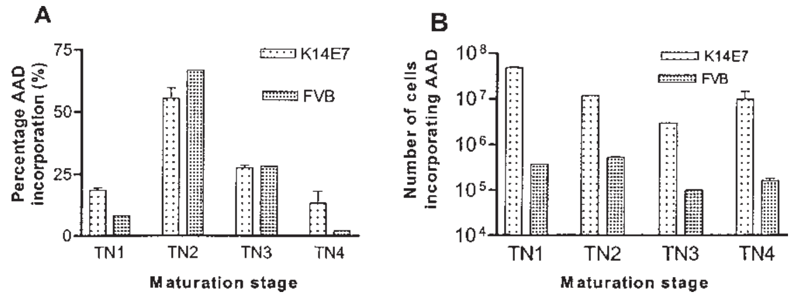

FIGURE 6 CD44 + CD25− (TN1) cells have an increased rate of apoptosis in the K14E7 tg mouse. Thymic cell preparations from 3 month old FVB and K14E7 mice were stained with CD44 PerCP and CD25 FITC and then sorted into TN1, TN2, TN3 and TN4 populations, which were then incubated with AAD. Uptake of this dye was used as a measure of apoptosis. (A) The most highly represented K14E7 TN population (CD44+ CD25−) had an increased level of AAD incorporation over the same population in the non-tg mouse. This increase in AAD incorporation was also shown for the final TN phase. (B) When the number of cells incorporating AAD was compared between tg and non-tg mice, there was a substantial increase in the number of dead and dying cells at all stages.

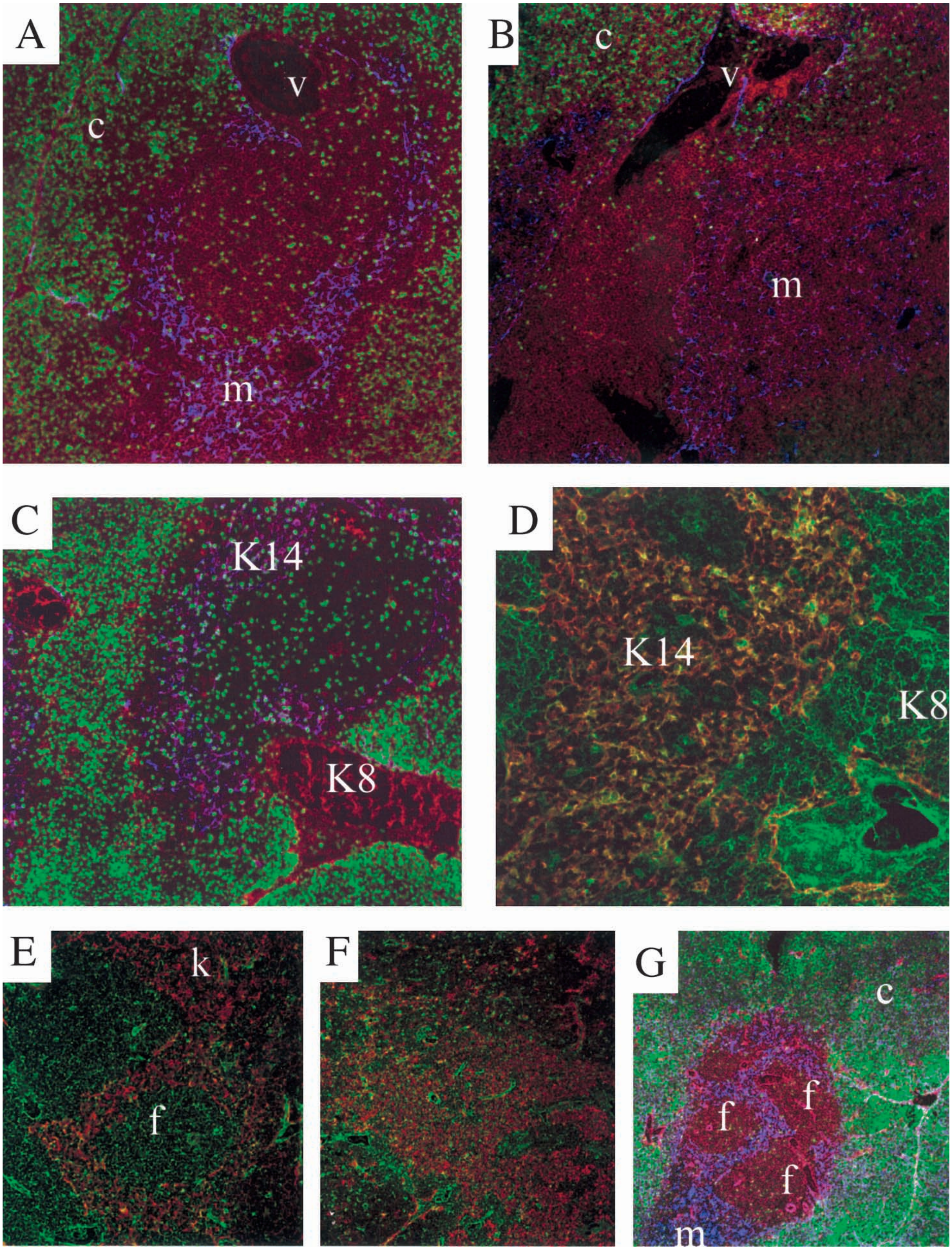

A
c
v
m
B
c
v
m
C
K14
K8
D
K14
K8
E
k
f
F
G
c
f
f
f
m compartment, suggesting that TN1 cells were being retained in this early compartment, becoming inactive and subsequently dying. Thus, expression of the K14E7 transgene affects maturation not only of late stage T cells but also of an early pre T compartment.

### Expanded Areas of Fibroblasts Lie Close to Densely K14 Positive Areas in the tg Mouse

The structure of the thymus was examined in K14E7 tg and non-tg animals. Various areas of the tg thymus ($K8^+$, $K14^+$, $CD3^+$ and fibroblast rich areas) were more sharply demarcated than in the non-tg thymus. Large blood vessels in the medullary area of the tg thymus were surrounded by accumulations of T cells, which were in turn surrounded by densely packed $K14^+$ TEC (Fig. 7A). $K14^+$ areas were more compact than in non-tg mice where $K14^+$ cells were scattered through the medullary stroma and were associated with intermediate-sized blood vessels. T cells were seen in smaller perivascular cuffs or evenly distributed throughout the stroma in the non-tg mouse thymus. BrdU incorporation was predominately in the $CD3^-$ $K14^-$ cortical stromal areas in the K14E7 mouse, as in the FVB mouse (Fig. 7A,B).

In the K14E7 tg mouse there were pockets of $K8^+$ cells which were quite separate from the $K14^+$ cells (Fig. 7C). The $K14^+$ areas and the cells they encircled showed significant BrdU incorporation, though not as intense as the BrdU staining in the $K8^-$ cortical areas. The pocketed K8 staining was not shown in non-tg mice (Fig. 7D), where there was significant overlap and interspersion of $K14^+$ and $K8^+$ cells.

Dense $K14^+$ cuffs of cells were seen perivasculary, adjacent to and surrounding areas staining strongly positive to MTS15, a fibroblast-specific marker (Fig. 7E). Fibroblasts accumulations also stained positive for the intracellular fibroblast marker, MTS7. In the non-tg mouse thymus, MTS7 and MTS15 staining was confined to scatterings throughout the medullary areas, staining most strongly at lobe junctions and in single layers perivascularly (Fig. 7F). These expanded fibroblast pockets surrounded smaller medullary blood vessels in the tg thymus and were themselves surrounded by $K14^+$ cells.

The MTS7/15 positive areas incorporated BrdU at a rate similar to that of the $K14^+$ cells in the transgenic thymus (Fig. 7G).

MTS6 staining, indicative of MHCII expression on cortical epithelium appeared similar in the tg and non-tg mice. BrdU incorporation in MTS6 staining areas was high in both, (data not shown) indicative of the high rate of immature T cell proliferation expected in the cortical area (Fehling and von Boehmer, 1997).

### DISCUSSION

Expression of the oncogenic HPV16E7 tg, targeted to thymic medullary epithelial cells by a K14 promoter, induces profound thymic hyperplasia in these transgenic mice. Epithelial cells constitute a major component of the thymic stroma and previous work has illustrated their importance both in thymus organogenesis and thymocyte maturation (Anderson *et al.*, 1993). The thymic niche model (Berzins *et al.*, 1999) suggests that the thymic stroma create a microenvironment capable of supporting the development and longevity of a certain number of T cells, any expansion of this microenvironment providing additional T cell niches and subsequently sustaining larger thymic T cell populations. Choreographed maturation of epithelia and thymocytes has been reported in several studies (Boyd *et al.*, 1993; van Ewijk *et al.*, 1994; Anderson *et al.*, 1996) and the expansion of early stage TEC is accompanied by enhanced support of the early stage T cell development in mice such as the K5-cyclin D1 and K5-HPV16E6/E7 tg mice (Klug *et al.*, 2000; Carraresi *et al.*, 2001). Targeted expression of the HPV 16E7 oncoprotein to squamous epithelium results in malignant transformation and hyperplasia of the epithelial cells (Arbeit *et al.*, 1994). In the K14E7 tg mice described here it could be thus expected that the expression of the oncogenic E7 protein in the thymic epithelium of the tg mice would induce a selective expansion of the mature $K14^+$ TEC compartment. Thymocyte development could then be more rapidly driven through the DP and SP stages due to enhanced availability of late-stage microenvironmental niches. The E7 mediated expansion of the medullary epithelium in our mice has led to not only

FIGURE 7 Immunofluorescence of frozen sections from 6 month old FVB and K14E7 thymii shows increased compartmentalization of keratin-staining, T cell and fibroblast areas in the transgenic thymus. Mice had received two doses of BrdU (5 and 1 h prior to thymus harvest) to allow an analysis of proliferation. BrdU-FITC incorporation (green) is most intense in the cortical area (c) of both K14E7 (A) and FVB (B) thymii (Magnigication × 100). Keratin 14-staining (blue) encircles area of mature T cells (CD3-red) which accumulate around large blood vessels. K14E7 mice have more densely packed K14+ areas, these areas incorporated BrdU at a higher level than do the more scattered K14+ areas of the FVB thymus. (C) Keratin 8 (red) compartmentalization is clearly shown as distinct from the K14+ areas (blue) of this K14E7 thymus (Magnification × 400). The majority of BrdU incorporation (green) occurs outside the K8 + /K14+ areas, however, a significant proportion is detected in K14+ areas and the areas encircled by K14+ cells. (D) The FVB thymus has less discrete bundles of K14+ (orange) and K8 + (green) epithelium. Extensive overlap occurs and the breakdown of architectural compartmentalization which occurs with age is apparent (Magnification, × 400). (E) Keratin (k) encircled fibroblast pockets (f) are dispersed through the K14E7 thymus. The use of fibroblast specific MTS15 antibody (green) on sections of the K14E7 thymus, shows these keratin-enclosed areas to clearly partitioned from the rest of the medulla. A pankeratin marker (red) is used in this section (Magnification × 100). (F) There is no similar compartmentalization of fibroblast accumulations in an equivalent preparation of FVB thymus. (G) The rate of cell cycling in these K14E7 fibroblast pockets was shown through the detection of BrdU incorporation (green). The keratin surrounding the fibroblast accumulations was shown to be keratin 14 (blue). MTS-7 (red) was used as an alternative fibroblast-specific antibody. BrdU incorporation was predominately confined to the cortical areas (c) with lower levels of proliferation occurring in these $K14^+$ or MTS-$7^+$ areas.

the support of mature T cells in the tg thymus but also to a relative reduction in their export to the periphery. Additional downstream events such as the expansion of a fibroblast population adjacent to the K14$^{+}$ epithelium and a skewing of the cells in the TN pre T cell compartment to the earlier TN stages were also observed.

There is controversy in the literature regarding the thymic pattern of K14-promoted transgenic antigen expression. Although endogenous K14 expression is restricted to medullary epithelium (Klug *et al.*, 1998), the K14 promoter has been shown to direct transgenic antigen expression to both the cortical and medullary thymic cells (McGargill *et al.*, 2000), to medullary and not cortical regions of the thymus (Burns *et al.*, 1999) and to thymic cortical but not medullary epithelium (Laufer *et al.*, 1996). This latter paper used a K14 promoter to target expression of a transgenic class II MHC antigen (I-A$^{b}$) to stratified squamous epithelium. Detection of the tg relied upon simultaneous expression of transgenic and endogeneous class II MHC in order that the tg I-A$^{b}$ was detected with their anti-class II heterodimer antibody. I-A$^{b}$ expression is not, however, homogeneous through TEC in the thymus (Surh *et al.*, 1992; Humblet *et al.*, 1994; Kasai *et al.*, 1996), MHC class II complexes being shown to be less stable in medullary TEC than in cortical TEC (Kasai *et al.*, 1996). Thus the absence of I-A detection in the medullary region of tg mice described in this paper may merely reflect the instability of medullary I-A$^{b}$ as opposed to definitively locating K14-promoted tg expression, which was not the purpose of the study. In contrast, K14-B7.1 tg mice which use the same K14 promoter used to create our K14E7 tg mice, showed tg B7.1 expression as being limited to the medullary epithelium (Burns *et al.*, 1999). The medullary staining pattern observed in these K14 B7-1 tg mice corresponded to the pattern of CD80 expression reported by others for normal thymic tissue (Nelson *et al.*, 1993; Degermann *et al.*, 1994). Our histological comparison of K14+ areas in the non-tg and K14E7 tg thymii showed a more densely packed medulla and more dense expression of medullary K14 in the tg mice. Secondary effects of K14-promoted E7 expression such as K14-encircled fibroblast pockets were also confined to the medullary area. Our data are thus consistent with E7 tg expression being targeted to medullary epithelium by our K14 promoter.

Recent papers have suggested that the progress of thymocytes through the maturation and selection process is determined not only upon the expression of certain cell surface markers but also upon the intensity of this expression (McKean *et al.*, 2001). We were able to correlate the level of T cell marker expression in the K14E7 mouse with thymic size and also the rate at which the cells died, divided and migrated to the periphery. Within the CD3$^{+}$ population the CD4 and CD8 markers were noticeably downregulated in the tg mouse from as early as 6 weeks old, the downregulation of CD44 in the TN population appearing only as the mice reached later life. The retained, predominately CD4$^{lo}$ cells were distinct from the recently described CD25$^{+}$ CD4$^{+}$ suppressor T cells (Takahashi *et al.*, 1998; Itoh *et al.*, 1999) in that they are CD3 bright and TCR$^{+}$, having already passed through TCR rearrangement and selection. Whilst a reduced level of expression of CD4 and CD8 on mature thymocytes is usually indicative of cells preparing for apoptotic death (Tiso and Gangemi, 1995), the non-cycling CD4$^{lo}$ and CD8$^{lo}$ cells retained in the K14E7 thymus had a reduced level of apoptosis. We have therefore shown that the downregulation of cell surface marker expression that normally accompanies programmed cell death is a distinct pathway that can occur independently of apoptotic death. It has been shown that the presence of mature CD4$^{+}$ cells plays a feedback role in the developmental signaling provided to the TN population (Fridkis-Hareli *et al.*, 1994; Mehr *et al.*, 1996). The observed accumulation of mature SP thymocytes in our K14E7 mice must, therefore, reach a critical number at which feedback inhibition begins to cause the accumulation of early TN stages at the expense numbers of cells in the later TN4 stage. The downregulation of CD44 in the TN1 and TN2 population is once again indicative of older, accumulating cells and can be considered a secondary event to SP cell accumulation in the older tg thymus.

Scollay and Godfrey proposed two opposing models of thymocyte exit from the thymus (Scollay and Godfrey, 1995): (Gabor *et al.*, 1997b) the conveyor belt model whereby thymocytes pass through obligatory maturation stages and are exported as SP cells expressing the full complement of maturational markers, and (Utsuyama *et al.*, 1991) the stochastic model whereby thymocytes spend a variable length of time in the medulla, acquiring phenotypic markers of maturation and leaving for the periphery at any stage during the maturational process. Our data tend to comply with the former model and the data of Chaffin and Perlmutter (1991) and Gabor *et al.* (1997b) which suggest that export from the thymus is the result of an active and regulated process dependent upon the cells having attained a certain stage of maturation and expressing appropriate levels of emigration-related signals. The higher level of expression of CD69 and CD5 in the K14E7 tg thymus is suggestive that the stromal signaling which controls post selection survival and exit from the thymus is being skewed by the expression of E7. It could be suggested that, due to the larger number of niches available for mature T cells, fewer SP T cells are receiving adequate stromal signaling and are less able to leave the tg thymus for the peripheral T cell pool, these cells merely wait to die in the thymus.

In our system, densely packed K14$^{+}$ epithelium, smaller pockets of K8$^{+}$ epithelium and unique fibroblast pockets were observed. A high level of compartmentalization of the thymic medulla into epithelial islets is characteristic of a juvenile thymus, the thymic structure generally deteriorating with age in normal mice (Rodewald *et al.*, 2001). A previous study comparing thymic development in the FVB strain to age- matched mice of other strains, showed that the FVB thymus undergoes premature thymic involution, showing loss of thymic architecture and loss of epithelial morphological differentiation 5 months earlier than other commonly used mouse strains (Nabarra *et al.*, 2001). The failure of the K14E7 thymus to involute and the significantly more defined thymic architecture in the adult tg mouse, suggests the presence of the transgene prevents the early aging shown by the FVB strain, maintaining a large cortical presence, clear compartmentalization and epithelial differentiation. Signals derived from K14 TEC are thus crucial to the control of age-related thymic involution and structural deterioration.

Since the niche model was proposed a more complex picture of thymic homeostatic regulation has emerged. Elements crucial to the control of T cell development within the thymic microenvironment include TEC- and fibroblast-derived cytokines such as IL7, SCF, TGF$\beta$ and TNFa which must be produced in adequate levels in order that cells may progress through crucial TN, DN and DP checkpoints. The stromal cell dependency of the thymocyte subsets varies markedly, with the $CD44^+$ TN stages being most dependent upon input from MHC class $II^+$ TEC and fibroblasts (Anderson *et al.*, 1997). Cytokines are also required to signal the upregulation of markers which will allow the cells to exit the thymus and be transported to the periphery (Diamond *et al.*, 1997; Gabor *et al.*, 1997b; Porcellini *et al.*, 1999). An E7-induced alteration in the thymic architecture and the maintenance of a "younger" thymic structure could be expected to alter the nature of the thymocyte-stromal cell interactions and the levels of cytokines to which the developing thymocytes are exposed. We postulate that, in the K14E7 mouse, the altered growth of $K14^+$-expressing epithelium under the direction of the oncogenic E7 protein leads to a change in the thymic architecture and composition such that cells important to the maturational process are altered in their relative abundance or contact with each other. This altered medullary composition is able to support DP to SP transitions and TCR mediated selection but appears to have a reduced ability to induce final export readiness or alternatively, produces a microenvironment containing levels of chemokines which result in thymocyte longevity and/or retention. Although there have been several reports demonstrating the affect of the cytokine mix in the stroma on the ability of thymocytes to progress normally through to mature SP cells (Porcellini *et al.*, 1999), this is the first report whereby an altered thymic microenvironment is able to effect the export of thymocytes to the peripheral T cell pool. As the thymocytes from these tg mice age without being exported they downregulate their markers but do not automatically enter the apoptotic pathway. Thus the immortalisation of a TEC population through the expression of a tg which is not expressed in thymocytes is able to prevent thymic involution. The number of cells in the thymus (thymocytes and stroma) increases dramatically without grossly disturbing the balance of T cells in the thymus or the structure or function of the peripheral immune system, with the exception of a deletion of CD8 T cells specific for E7 (Frazer *et al.*, 1998). The expansion of the K14E7 thymic T cell population was reflected in a 50% increase in the peripheral T cell population. This concurs with the findings of Berzins *et al.* (1999) whereby severely hyperthymic mice were able to overcome the usual homeostatic control over the number of peripheral T cells.

The degree to which the failure to involute, the accumulation of mature thymic T cells and other ageing-related events, such as the age-related loss of immune function, are interdependent can thus be explored with our K14E7 tg mouse model. Preliminary data has shown that the observed downregulation of CD4 and CD8 expression and thymic hypertrophy is due to the secretion of soluble factors by the K14E7 TEC population (paper in preparation). Further work is underway to isolate these cellular products and to determine the nature of the thymocyte/stromal cell interactions involved in the abnormal maturational outcomes observed in the K14E7 tg thymus.

### *Acknowledgements*

The assistance of Dale Godfrey and Adam Uldrich for the intrathymic labeling was greatly appreciated. We are also grateful for the technical assistance of Olivia White for the measurement of E7 mRNA. We thank Nick Saunders of C.I.C.R. and all members of the Boyd laboratory, Monash University, for useful discussion. This work was supported by the National Health and Medical Research Council of Australia.

### *References*

Anderson, G., Jenkinson, E.J., Moore, N.C. and Owen, J.J. (1993) "MHC class II-positive epithelium and mesenchyme cells are both required for T-cell development in the thymus", *Nature* **362**, 70.

Anderson, G., Moore, N.C., Owen, J.J. and Jenkinson, E.J. (1996) "Cellular interactions in thymocyte development", *Annu. Rev. Immunol.* **14**, 73.

Anderson, G., Anderson, K.L., Tchilian, E.Z., Owen, J.J. and Jenkinson, E.J. (1997) "Fibroblast dependency during early thymocyte development maps to the CD25+ CD44+ stage and involves interactions with fibroblast matrix molecules", *Eur. J. Immunol.* **27**, 1200.

Arbeit, J.M., Munger, K., Howley, P.M. and Hanahan, D. (1994) "Progressive squamous epithelial neoplasia in K14-human papillomavirus type 16 transgenic mice", *J. Virol.* **68**, 4358.

Berzins, S.P., Godfrey, D.I., Miller, J.F. and Boyd, R.L. (1999) "A central role for thymic emigrants in peripheral T cell homeostasis", *Proc. Natl Acad. Sci. USA* **96**, 9787.

Boyd, R.L., Tucek, C.L., Godfrey, D.I., Izon, D.J., Wilson, T.J., Davidson, N.J., Bean, A.G., Ladyman, H.M., Ritter, M.A. and Hugo, P. (1993) "The thymic microenvironment", *Immunol. Today* **14**, 445.

Burns, R.P. Jr., Nasir, A., Haake, A.R., Barth, R.K. and Gaspari, A.A. (1999) "B7-1 overexpression by thymic epithelial cells results in transient and long- lasting effects on thymocytes and peripheral T helper cells but does not result in immunodeficiency", *Cell Immunol.* **194**, 162.

Carraresi, L., Tripodi, S.A., Mulder, L.C., Bertini, S., Nuti, S., Schuerfeld, K., Cintorino, M., Bensi, G., Rossini, M. and Mora, M. (2001) "Thymic hyperplasia and lung carcinomas in a line of mice transgenic for keratin 5-driven HPV16 E6/E7 oncogenes", *Oncogene* **20**, 8148.

Chaffin, K.E. and Perlmutter, R.M. (1991) "A pertussis toxin-sensitive process controls thymocyte emigration", *Eur. J. Immunol.* **21**, 2565.

Davies, R., Hicks, R., Crook, T., Morris, J. and Vousden, K. (1993) "Human papillomavirus type 16 E7 associates with a histone H1 kinase and with p107 through sequences necessary for transformation", *J. Virol.* **67**, 2521.

Degermann, S., Surh, C.D., Glimcher, L.H., Sprent, J. and Lo, D. (1994) "B7 expression on thymic medullary epithelium correlates with epithelium- mediated deletion of V beta 5+ thymocytes", *J. Immunol.* **152**, 3254.

Demers, G.W., Espling, E., Harry, J.B., Etscheid, B.G. and Galloway, D.A. (1996) "Abrogation of growth arrest signals by human papillomavirus type 16 E7 is mediated by sequences required for transformation", *J. Virol.* **70**, 6862.

Diamond, R.A., Ward, S.B., Owada-Makabe, K., Wang, H. and Rothenberg, E.V. (1997) "Different developmental arrest points in RAG-2 −/− and SCID thymocytes on two genetic backgrounds: developmental choices and cell death mechanisms before TCR gene rearrangement", *J. Immunol.* **158**, 4052.

van Ewijk, W., Shores, E.W. and Singer, A. (1994) "Crosstalk in the mouse thymus", *Immunol. Today* **15**, 214.

Fehling, H.J. and von Boehmer, H. (1997) "Early alpha beta T cell development in the thymus of normal and genetically altered mice", *Curr. Opin. Immunol.* **9**, 263.

Frazer, I.H., Fernando, G.J., Fowler, N., Leggatt, G.R., Lambert, P.F., Liem, A., Malcolm, K. and Tindle, R.W. (1998) "Split tolerance to a viral antigen expressed in thymic epithelium and keratinocytes", *Eur. J. Immunol.* **28**, 2791.

Frazer, I.H., De Kluyver, R., Leggatt, G.R., Guo, H.Y., Dunn, L., White, O., Harris, C., Liem, A. and Lambert, P. (2001) "Tolerance or immunity to a tumor antigen expressed in somatic cells can be determined by systemic proinflammatory signals at the time of first antigen exposure", *J. Immunol.* **167**, 6180.

Fridkis-Hareli, M., Mehr, R., Abel, L. and Globerson, A. (1994) "Developmental interactions of CD4 T cells and thymocytes: age-related differential effects", *Mech. Ageing Dev.* **73**, 169.

Gabor, M.J., Godfrey, D.I. and Scollay, R. (1997a) "Recent thymic emigrants are distinct from most medullary thymocytes", *Eur. J. Immunol.* **27**, 2010.

Gabor, M.J., Scollay, R. and Godfrey, D.I. (1997b) "Thymic T cell export is not influenced by the peripheral T cell pool", *Eur. J. Immunol.* **27**, 2986.

Godfrey, D.I., Izon, D.J., Tucek, C.L., Wilson, T.J. and Boyd, R.L. (1990) "The phenotypic heterogeneity of mouse thymic stromal cells", *Immunology* **70**, 66.

Gray, D.H., Chidgey, A.P. and Boyd, R.L. (2002) "Analysis of thymic stromal cell populations using flow cytometry", *J. Immunol. Methods* **260**, 15.

Herber, R., Liem, A., Pitot, H. and Lambert, P.F. (1996) "Squamous epithelial hyperplasia and carcinoma in mice transgenic for the human papillomavirus type 16 E7 oncogene", *J. Virol.* **70**, 1873.

Hollander, G.A., Wang, B., Nichogiannopoulou, A., Platenburg, P.P., van Ewijk, W., Burakoff, S.J., Gutierrez-Ramos, J.C. and Terhorst, C. (1995) "Developmental control point in induction of thymic cortex regulated by a subpopulation of prothymocytes", *Nature* **373**, 350.

Humblet, C., Rudensky, A. and Kyewski, B. (1994) "Presentation and intercellular transfer of self antigen within the thymic microenvironment: expression of the E alpha peptide-I-Ab complex by isolated thymic stromal cells", *Int. Immunol.* **6**, 1949.

Itoh, M., Takahashi, T., Sakaguchi, N., Kuniyasu, Y., Shimizu, J., Otsuka, F. and Sakaguchi, S. (1999) "Thymus and autoimmunity: production of CD25 + CD4+ naturally anergic and suppressive T cells as a key function of the thymus in maintaining immunologic self-tolerance", *J. Immunol.* **162**, 5317.

Kasai, M., Hirokawa, K., Kajino, K., Ogasawara, K., Tatsumi, M., Hermel, E., Monaco, J.J. and Mizuochi, T. (1996) "Difference in antigen presentation pathways between cortical and medullary thymic epithelial cells", *Eur. J. Immunol.* **26**, 2101.

Klug, D.B., Carter, C., Crouch, E., Roop, D., Conti, C.J. and Richie, E.R. (1998) "Interdependence of cortical thymic epithelial cell differentiation and T-lineage commitment", *Proc. Natl Acad. Sci. USA* **95**, 11822.

Klug, D.B., Crouch, E., Carter, C., Coghlan, L., Conti, C.J. and Richie, E.R. (2000) "Transgenic expression of cyclin D1 in thymic epithelial precursors promotes epithelial and T cell development", *J. Immunol.* **164**, 1881.

Laufer, T.M., DeKoning, J., Markowitz, J.S., Lo, D. and Glimcher, L.H. (1996) "Unopposed positive selection and autoreactivity in mice expressing class II MHC only on thymic cortex", *Nature* **383**, 81.

McGargill, M.A., Derbinski, J.M. and Hogquist, K.A. (2000) "Receptor editing in developing T cells", *Nat. Immunol.* **1**, 336.

McKean, D.J., Huntoon, C.J., Bell, M.P., Tai, X., Sharrow, S., Hedin, K.E., Conley, A. and Singer, A. (2001) "Maturation versus death of developing double-positive thymocytes reflects competing effects on Bcl-2 expression and can be regulated by the intensity of CD28 costimulation", *J. Immunol.* **166**, 3468.

Mehr, R., Perelson, A.S., Fridkis-Hareli, M. and Globerson, A. (1996) "Feedback regulation of T cell development in the thymus", *J. Theor. Biol.* **181**, 157.

Nabarra, B., Mulotte, M., Casanova, M., Godard, C. and London, J. (2001) "Ultrastructural study of the FVB/N mouse thymus: presence of an immature epithelial cell in the medulla and premature involution", *Dev. Comp. Immunol.* **25**, 231.

Nelson, A.J., Hosier, S., Brady, W., Linsley, P.S. and Farr, A.G. (1993) "Medullary thymic epithelium expresses a ligand for CTLA4 *in situ* and *in vitro*", *J. Immunol.* **151**, 2453.

Penit, C., Lucas, B. and Vasseur, F. (1995) "Cell expansion and growth arrest phases during the transition from precursor (CD4 − 8−) to immature (CD4 + 8+) thymocytes in normal and genetically modified mice", *J. Immunol.* **154**, 5103.

Porcellini, S., Panigada, M. and Grassi, F. (1999) "Molecular and cellular aspects of induced thymus development in recombinase-deficient mice", *Eur. J. Immunol.* **29**, 2476.

Rodewald, H.R., Kretzschmar, K., Swat, W. and Takeda, S. (1995) "Intrathymically expressed c-kit ligand (stem cell factor) is a major factor driving expansion of very immature thymocytes *in vivo*", *Immunity* **3**, 313.

Rodewald, H.R., Paul, S., Haller, C., Bluethmann, H. and Blum, C. (2001) "Thymus medulla consisting of epithelial islets each derived from a single progenitor", *Nature* **414**, 763.

Roop, D.R., Cheng, C.K., Titterington, L., Meyers, C.A., Stanley, J.R., Steinert, P.M. and Yuspa, S.H. (1984) "Synthetic peptides corresponding to keratin subunits elicit highly specific antibodies", *J. Biol. Chem.* **259**, 8037.

Schreiber, L., Eshel, I., Meilin, A., Sharabi, Y. and Shoham, J. (1991) "Analysis of thymic stromal cell subpopulations grown *in vitro* on extracellular matrix in defined medium. III. Growth conditions of human thymic epithelial cells and immunomodulatory activities in their culture supernatant", *Immunology* **74**, 621.

Scollay, R. and Godfrey, D.I. (1995) "Thymic emigration: conveyor belts or lucky dips?", *Immunol. Today* **16**, 268.

Scollay, R.G., Butcher, E.C. and Weissman, I.L. (1980) "Thymus cell migration. Quantitative aspects of cellular traffic from the thymus to the periphery in mice", *Eur. J. Immunol.* **10**, 210.

Surh, C.D., Ernst, B. and Sprent, J. (1992) "Growth of epithelial cells in the thymic medulla is under the control of mature T cells", *J. Exp. Med.* **176**, 611.

Takahashi, T., Kuniyasu, Y., Toda, M., Sakaguchi, N., Itoh, M., Iwata, M., Shimizu, J. and Sakaguchi, S. (1998) "Immunologic self-tolerance maintained by CD25 + CD4+ naturally anergic and suppressive T cells: induction of autoimmune disease by breaking their anergic/suppressive state", *Int. Immunol.* **10**, 1969.

Thoman, M.L. (1995) "The pattern of T lymphocyte differentiation is altered during thymic involution", *Mech. Ageing Dev.* **82**, 155.

Tindle, R.W., Herd, K., Doan, T., Bryson, G., Leggatt, G.R., Lambert, P., Frazer, I.H. and Street, M. (2001) "Nonspecific down-regulation of CD8+ T-cell responses in mice expressing human papillomavirus type 16 E7 oncoprotein from the keratin-14 promoter", *J. Virol.* **75**, 5985.

Tiso, M., Gangemi, R., Bargellesi Severi, A., Pizzolitto, S., Fabbi, M. and Risso, A. (1995) "Spontaneous apoptosis in human thymocytes", *Am. J. Pathol.* **147**, 434.

Utsuyama, M., Kasai, M., Kurashima, C. and Hirokawa, K. (1991) "Age influence on the thymic capacity to promote differentiation of T cells: induction of different composition of T cell subsets by aging thymus", *Mech. Ageing Dev.* **58**, 267.

